# ROOTS TO GROW AND WINGS TO FLY: EDUCATING MEDICINE AND PHARMACY TRAINEES ON VIONE MEDICATION DEPRESCRIBING METHODOLOGY

**DOI:** 10.1093/geroni/igad104.0903

**Published:** 2023-12-21

**Authors:** Saraswathy Battar, Michael Silverman

## Abstract

An aging world population translates to more chronic illnesses, more medications with potential and relevant cumulative burdens such as non-compliance, adverse outcomes, prescription cascades, confusion, compromised patient safety and quality of life standards. VIONE stands for the 5 filters that categorize medications as Vital, Important, Optional, Not indicated, Every medication has an indication. This is an electronic health record methodology currently implemented in 122 VA facilities across the nation. Physicians and pharmacists review medications, prioritize medications that merit deprescribing interventions, deprescribe through shared decision making and monitor outcomes. In the VA system, within 7 years, we successfully deprescribed over 1.4 million inappropriate medications impacting over 600,000 veterans, used by over 14000 medical providers, with an annualized cost savings of ~ 132 million US dollars. We incorporate VIONE deprescribing education while training our pharmacy residents, medical students, residents and fellows rotating at our VA Medical Center through case based, bed side, didactic and team based multi media learning strategies . The engagement, feedback, demonstrated formal and informal responses remain very promising. Capturing the attention of trainees in their formative years deepens their roots as they spread their wings into practicing safe medication prescribing practices that are current, cutting edge, evidence based and empowering as we train the trainers. We envision medication deprescribing to be part of future academic core curriculum. Our Interdisciplinary team will present compelling data, experiences and outcomes stemming from education of trainees regarding safe medication optimization and deprescribing practices utilizing VIONE methodology that won national and international acclaim.

